# Base of Support, Step Length and Stride Width Estimation during Walking Using an Inertial and Infrared Wearable System

**DOI:** 10.3390/s23083921

**Published:** 2023-04-12

**Authors:** Rachele Rossanigo, Marco Caruso, Stefano Bertuletti, Franca Deriu, Marco Knaflitz, Ugo Della Croce, Andrea Cereatti

**Affiliations:** 1 Department of Biomedical Sciences, University of Sassari, 07100 Sassari, Italy; sbertuletti@uniss.it (S.B.); deriuf@uniss.it (F.D.); dellacro@uniss.it (U.D.C.); 2 PolitoBIOMed Lab—Biomedical Engineering Lab, Politecnico di Torino, 10129 Torino, Italy; marco.caruso@polito.it (M.C.); marco.knaflitz@polito.it (M.K.); 3 Department of Electronics and Telecommunications, Politecnico di Torino, 10129 Torino, Italy; 4 Unit of Endocrinology, Nutritional and Metabolic Disorders, AOU Sassari, 07100 Sassari, Italy

**Keywords:** base of support, inertial sensors, infrared time-of-flight distance sensors, wearable system, dynamic stability, gait analysis

## Abstract

The analysis of the stability of human gait may be effectively performed when estimates of the base of support are available. The base of support area is defined by the relative position of the feet when they are in contact with the ground and it is closely related to additional parameters such as step length and stride width. These parameters may be determined in the laboratory using either a stereophotogrammetric system or an instrumented mat. Unfortunately, their estimation in the real world is still an unaccomplished goal. This study aims at proposing a novel, compact wearable system, including a magneto-inertial measurement unit and two time-of-flight proximity sensors, suitable for the estimation of the base of support parameters. The wearable system was tested and validated on thirteen healthy adults walking at three self-selected speeds (slow, comfortable, and fast). Results were compared with the concurrent stereophotogrammetric data, used as the gold standard. The root mean square errors for the step length, stride width and base of support area varied from slow to high speed between 10–46 mm, 14–18 mm, and 39–52 cm^2^, respectively. The mean overlap of the base of support area as obtained with the wearable system and with the stereophotogrammetric system ranged between 70% and 89%. Thus, this study suggested that the proposed wearable solution is a valid tool for the estimation of the base of support parameters out of the laboratory.

## 1. Introduction

**Figure 1:**
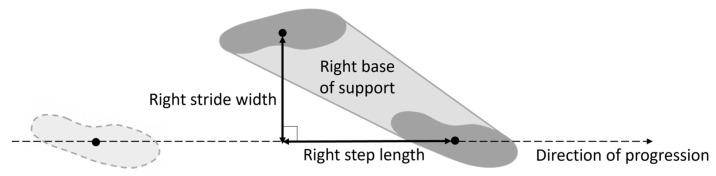
Gait parameters related to a right base of support, with reference to a right gait cycle. The position of a footprint is approximated by its centroid. The direction of progression for the right gait cycle is identified by the line connecting the centroids of two consecutive right footprints.

The role of the base of support (BoS) is crucial in the investigation of the dynamic stability during walking [1]. With reference to normal gait, the right BoS is defined as the area enclosed by the outer edges of the footprints with the right foot ahead the left one [2], and it is associated with the right *step length* and the right *stride width* (Figure 1). Given the distance between two consecutive footprints, *step length* and *stride width* are defined as the projections of the above-mentioned distance, respectively, on the direction of progression (identified by two consecutive homolateral footprints) and on the line perpendicular to it [3]. Several studies have investigated the correlations between BoS-related parameters and margin of stability, balance, and risk of falling both in normal and pathological gait [4,5,6,7,8].

The BoS can be obtained in the laboratory from the direct measures of the feet position of a stereophotogrammetric system (SP) or of an instrumented mat [9,10,11]. Much more difficult is to obtain an accurate description of the BoS out of the laboratory and in the real world.

To date, magneto-inertial measurement units (MIMUs) attached to the feet are the most effective wearable technology for out-of-laboratory gait analysis [12].

MIMU-based methods have been successfully used for the estimation of spatio-temporal parameters such as stride duration and length [13,14]; however, the use of inertial sensing technology alone cannot provide information about the relative position of the two feet, and consequently, BoS-related parameters.

In the literature, different ancillary technologies have been proposed to overcome the intrinsic MIMU’s limitation by integrating other types of sensors enabling the estimation of the relative feet position.

The most direct solution to continuously measure the inter-foot distance is to integrate MIMUs with either ultrasound sensors [15,16,17] or foot-worn cameras [18,19]. These systems can provide accurate foot trajectories, but they are quite cumbersome and therefore not suitable for both clinical and real-world applications.

To reduce the system size, Trojaniello et al. [20] proposed a system integrating MIMUs and a light intensity infrared proximity sensor which embeds a transmitter and a receiver in the same chip. However, infrared proximity sensors do not provide the inter-foot distance, but rather the distance between the infrared emitter and any reflecting target. In addition, their performance may vary due to changes in the environmental conditions, such as reflectance and color of the target surface [21].

A further improvement was obtained by using infrared time-of-flight proximity sensors, which guarantee a good accuracy regardless of the environmental conditions [21]. By instrumenting a single foot with the latter system, Bertuletti et al. [22] developed a method for step detection and relevant inter-foot distance estimation.

In summary, among the various solutions proposed in the literature, some methods limited the analysis to the estimation of inter-foot distance [20,22], some others focused on the reconstruction of feet trajectories for pedestrian navigation purposes [15,18,19], and finally, methods based on ultrasound technology provided *step length*, *stride width* [16], and margin of stability [17], but not the *BoS area*.

In the present study, we propose an original wearable system which integrates miniaturized infrared time-of-flight sensors with inertial sensing for the estimation of BoS parameters. Its main advantage with respect to existing solutions is that only one foot is instrumented, thus improving wearability and portability. The position of the non-instrumented foot during stance is estimated from the distance data recorded during the swing phase of the instrumented foot.

The validation of the proposed system was carried out on thirteen healthy subjects walking at three self-selected speeds (slow, comfortable, and fast) and results were compared in terms of *BoS area*, *step length*, and *stride width* to those obtained from SP, considered as the gold standard.

## 2. Materials and Methods

### 2.1. System Overview

**Figure 2:**
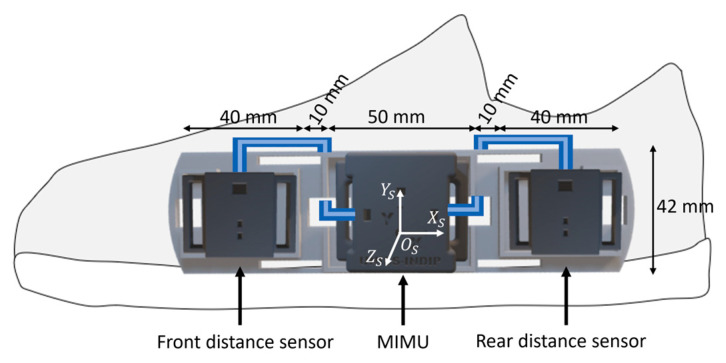
Wearable system: a magneto-inertial unit and two infrared time-of-flight distance sensors are cabled (in blue) and fixed to a custom 3D-printed rigid support attached to the medial side of a shoe. The size of the wearable system (sensors and rigid support) is 155 mm × 42 mm × 22 mm and the overall weight is ~40 g. The MIMU coordinate system (CS_S_) is depicted in white.

The proposed system included an MIMU and two distance sensors (DS) connected to the inertial module via cable [22,23]. All sensors were embedded in a custom 3D-printed rigid support with known geometry, fixed to the medial side of a shoe through two thin straps, based on the experimental setup proposed in a previous study [22] (Figure 2).

The MIMU (mod. LSMDSO and mod. LIS2MDL, STMicroelectronics, Switzerland; 3D accelerometer: range ±16 g; 3D gyroscope: range ±2000 dps; 3D magnetometer: range ±50 Gauss; sampling frequency: 100 Hz) was calibrated following the methods proposed by [24,25].

The DS (mod. VL6180X, STMicroelectronics, Switzerland; distance: range 0–200 mm; sampling frequency: 50 Hz) reduced the sensor’s dimensions (4.8 mm × 2.8 mm × 1.0 mm), combined the receiver and the transmitter in the same chip, and guaranteed low power consumption (~2–5 mA) and an accuracy independent from environmental conditions. The infrared time-of-flight technology provides an estimate of the distance to the target by measuring the phase shift between the radiated and the reflected infrared waves. DSs were calibrated with a custom 3D-printed cylinder which imposed a known distance between the sensor and the target, so that the offset could be estimated and removed. Distance data were linearly interpolated and resampled at 100 Hz.

Recorded magneto-inertial and distance data were stored onboard and the communication between the laptop and the MIMUs was based on the Bluetooth low energy technology.

### 2.2. Description of the Method for Base of Support Estimation

**Figure 3:**
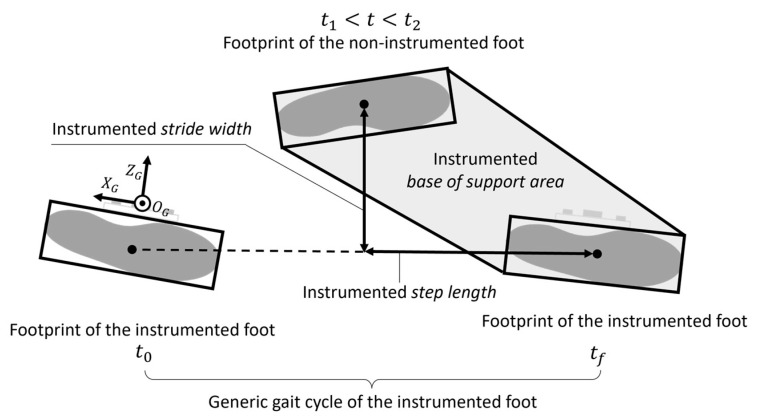
Base of support parameters associated with a generic gait cycle of the instrumented (right) foot. The instrumented base of support is the area surrounded by the outer edges of the footprint of the non-instrumented foot and the consecutive contralateral one. *t*_0_ and *t_f_* are consecutive flat-foot instants of the instrumented foot. *t*_1_–*t*_2_ is the portion of the flat-foot phase of the non-instrumented foot in which the distance sensors record inter-foot distances. The chosen global coordinate system (CS_G_) for the considered gait cycle is the MIMU coordinate system at *t*_0_.

**Figure 4:**
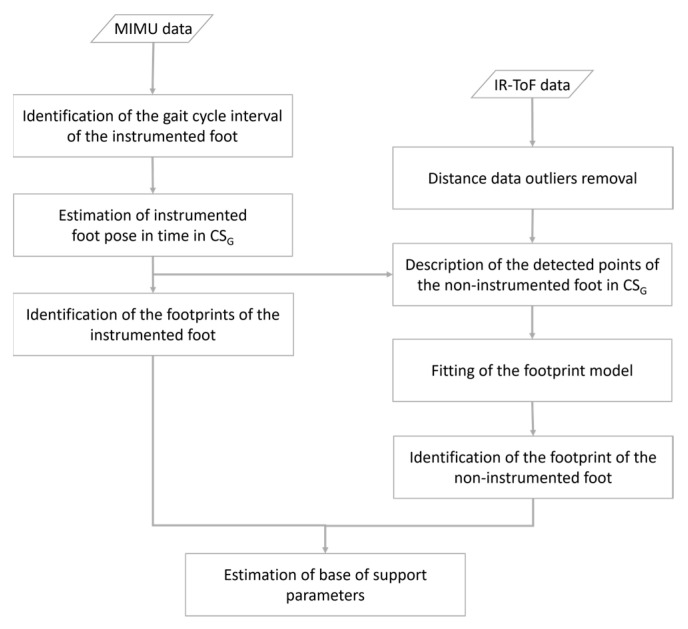
Flowchart of the methods implemented to estimate the base of support parameters from MIMU and infrared time-of-flight (IR-ToF) distance data with respect to the global coordinate system (CS_G_).

By exploiting gait cyclicity, the procedure for the estimation of the BoS is presented with reference to a generic gait cycle of the instrumented foot, which was arbitrarily chosen to be the right one. Thus, hereby we refer to the right BoS as instrumented BoS. The estimation of the instrumented *BoS area*, *step length*, and *stride width* requires one to determine position and orientation of two right and one left footprints with respect to the same global coordinate system (CS_G_) (Figure 3). To this purpose, the following actions were implemented: (1) identification of the gait cycle interval of the instrumented foot, (2) estimation of the position and orientation (pose) of the instrumented foot, (3) identification of the footprints of the instrumented foot, (4) identification of the footprint of the non-instrumented foot, and (5) computation of BoS-related parameters (Figure 4).

#### 2.2.1. Identification of the Gait Cycle Interval of the Instrumented Foot

The gait cycle interval was defined as the interval of time between two consecutive flat-foot instants, *t*_0_ and *t_f_*, of the instrumented foot with the lowest kinetic energy. The stance phases were identified through peak detection on the approximated foot mediolateral angular velocities and anteroposterior accelerations [13,26]. The flat-foot instants were searched within the relevant stance phase of the instrumented foot using a parametric zero-velocity detector based on angular velocities [27,28].

#### 2.2.2. Estimation of the Instrumented Foot Pose in the Global Coordinate System CS_G_

The coordinate system fixed with the instrumented foot (CS_S_) was made to coincide with the coordinate system embedded with the sensor axes of the MIMU (Figure 2). The orientation of CS_S_ with respect to the Earth magnetic-gravity reference system (CS_E_), expressed by the rotation matrix RSE(t), was obtained from the recorded magneto-inertial data using an optimized complementary filter [28,29,30,31].

The foot acceleration in CS_E_, aE(t), was computed by removing the gravity contribution to the recorded accelerations:

(1) aE(t)=RSE·fS(t)−g

where fS(t) is the specific force measured by the accelerometer in CS_S_ and ***g*** is the gravity vector.

A global coordinate system CS_G_ was made to coincide with CS_S_ at the beginning of the gait cycle (*t*_0_) after realignment with the gravity, so that the *y*-axis of CS_G_ was perpendicular to the ground (Figure 3). The origin O_G_ was set to the ground plane. 

Then, aE(t) was expressed in CS_G_:

(2) aG(t)=REG·aE(t)

where REG is the rotation matrix from CS_E_ to CS_G_.

Finally, the foot trajectory, represented by the position of the origin O_S_ of CS_S_, pOSG(t), was computed by double integrating aG(t) between *t*_0_ and *t_f_*:

(3) pOSG(t)=pOSG(t0)+∬t0taGτdτ t∈[t0,tf]

To reduce the drift, an optimal filtering of accelerations and a direct and reverse integration technique for the velocity estimation were implemented [28,32]. The initial boundary conditions of velocity and displacement were set to zero.

The foot orientation in time with respect to CS_G_ was described by the rotation matrix from CS_S_ to CS_G_, RSGt, computed as follows:

(4) RSGt=REG·RSE(t) t∈[t0,tf]

The foot pose in time with respect to CS_G_ was described by the transformation matrix from CS_S_ to CS_G_, TSGt, computed as follows:

(5) TSGt=RSGtpOSGt01 t∈t0,tf

where pOSGt is the translation vector of the foot position (i.e., origin O_S_) from the origin O_G_.

#### 2.2.3. Identification of the Footprints of the Instrumented Foot

The footprint was defined by the outer borders of the contact region between the foot insole and the ground plane during the relevant flat-foot instant. For simplicity, the footprint was approximated to a rectangle with vertices *V_i_* (*i* = 1, 2, 3, 4), and length *L* and width *W* equal to the measured subject’s shoe sizes.

**Figure 5:**
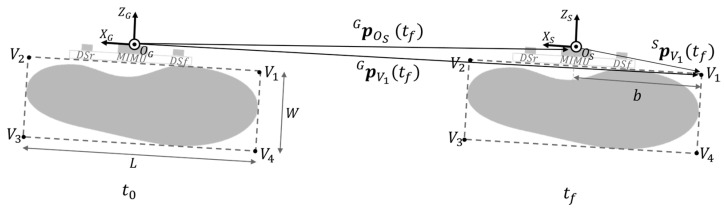
The footprints of the instrumented foot (dashed rectangles) are shown for two consecutive flat-foot instants (*t*_0_ and *t_f_*). The foot shape is approximated with a rectangle of vertices *V*_1_, *V*_2_, *V*_3,_ and *V*_4_. Shoe width *W* ($\overset{-}{V_{1}V_{4}}$), shoe length *L* $(\overset{-}{V_{3}V_{4}}$), and distance *b* ($\overset{-}{O_{S}V_{1}}$) were measured with a ruler. Position vectors of the vertices in CS_S_ were expressed by means of simple geometrical rules (e.g., pV1S=−b,0,0T). Then, the position vector of the vertices of the footprint pViG(t) at *t*_0_ and *t_f_* are expressed in the global coordinate system CS_G_.

The time-invariant position of each vertex *V_i_* of the rectangle in the CS_S_, pViS (*i* = 1, 2, 3, 4), was identified based on *L*, *W*, and distance *b* as obtained during a calibration procedure (Figure 5).

The positions of each vertex *V_i_* (*i* = 1, 2, 3, 4) expressed in CS_G_, pViG(t) (*i* = 1, 2, 3, 4), at *t*_0_ and *t_f_* can be computed as follows:

(6) pViGt01=TSGt0·pViS1 i=1,2,3,4

(7) pViG(tf)1=TSGtf·pViS1 i=1,2,3,4

Footprints are then defined by the x_G_-z_G_ components of pViG (*i* = 1, 2, 3, 4) at *t*_0_ and *t_f_*.

#### 2.2.4. Identification of the Footprint of the Non-Instrumented Foot

The footprint of the non-instrumented foot was determined based on the knowledge of the instrumented foot pose and the data recorded by the two DS. In fact, during the swing phase of the instrumented foot, occurring during the stance phase of the non-instrumented foot, the two feet face each other, and the DS readings are different from zero and equal to the distance between medial shoe surfaces.

**Figure 6:**
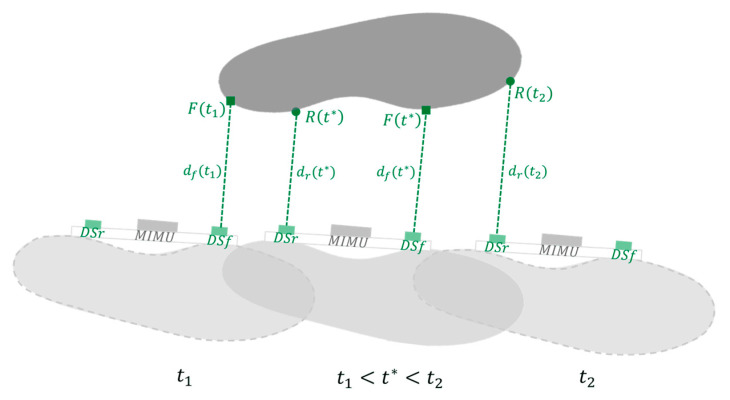
The instrumented foot (light grey) is in swing phase and faces the non-instrumented foot (dark grey), which is still during its stance phase. Between *t*_1_ and *t*_2_, the front and rear distance sensors (DS_f_ and DS_r_) record distances *d_f_*(*t*) and *d_r_*(*t*) by detecting points *F*(*t*) (green squares) and *R*(*t*) (green dots) of the medial side of the non-instrumented foot.

Let *t*_1_ be the timing of the first reading different from zero obtained from the DS attached to the front portion of the instrumented foot (DS_f_) and equal to the distance $d_{f}\left(t_{1}\right)$ between DS_f_ and the point $F(t_{1})$ of the medial side of the non-instrumented foot. Let *t*_2_ be the timing of the last reading different from zero of DS attached to the rear portion of the instrumented foot (DS_r_) and equal to the distance $d_{r}\left(t_{2}\right)$ between DS_r_ and the point $R(t_{2})$ of the medial side of the non-instrumented foot (Figure 6).

Let t* be a generic instant of time between *t*_1_ and *t*_2_, during which the non-instrumented foot was still, when both DS_f_ and DS_r_ could measure non-zero values equal to distances $d_{f}\left(t^{*}\right)$ and $d_{r}\left(t^{*}\right)$ between DS_f_ and DS_r_ and the points $F(t^{*})$ and $R(t^{*})$ of the medial side of the non-instrumented foot, respectively (Figure 6).

**Figure 7:**
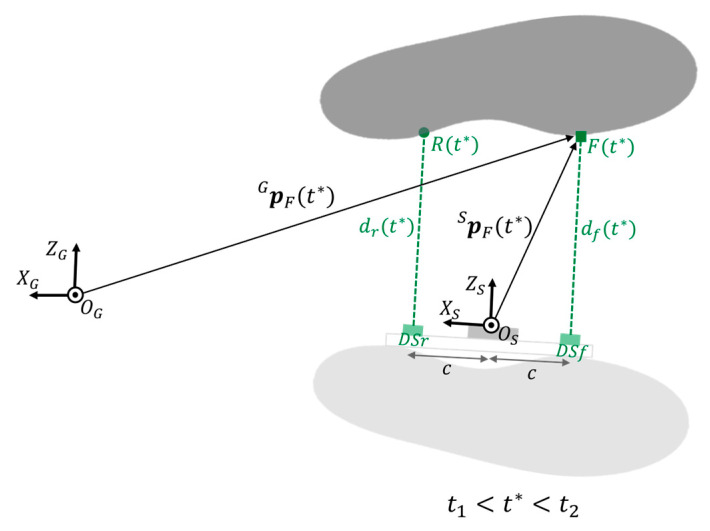
During *t*_1_ < t* < *t*_2_ the non-instrumented foot (dark grey) is in stance phase, while the instrumented foot (light grey) is in swing phase. $d_{f}$(t*) and $d_{r}$(t*) are the distances recorded by the front and rear distance sensors (DSr and DSf) at t*. *R*(t*) and *F*(t*) are the points of the medial side of the non-instrumented foot detected by the DS. c is the distance between each DS and the origin O_S_. For instance, the position of point *F*(t*) with respect to CS_S_, pFS(t*) is calculated by means of geometrical rules exploiting $d_{f}$(t*) and *c*. Then, the position vector pFG(t*) is computed to describe the point *F*(t*) with respect to the global coordinate system CS_G_.

The positions of the points $F(t^{*})$ and $R\left(t^{*}\right)$ with respect to CS_S_, pFSt* and pRSt*, were computed as follows:

(8) pFSt*=−c0dft* t*∈t1,t2

(9) pRSt*=c0drt* t*∈t1,t2

where $d_{f}(t^{*})$ and $d_{r}(t^{*})$ are the distances recorded at t* by DS_f_ and DS_r_, respectively, and *c* is the constant distance measured between each DS and O_S_ (Figure 7).

Then, the detected points were expressed with respect to CS_G_:

(10) pFGt1=TSG·pFSt1 t∈[t1,t2]

(11) pRGt1=TSG·pRSt1 t∈[t1,t2]

The projection of points F(t) and R(t) to the ground plane (x_G_-z_G_), pFxG, pFzG,pRxG, and pRzG, were then stored in the matrix ***M***:

(12) M=pFxGt1pFzGt1pFxGt1+∆tpFzGt1+∆tpRxGt1+∆tpRzGt1+∆t......pRxGt2pRzGt2

where ***M*** is a Nx2 matrix with N equal to the total number of the recorded points of the non-instrumented foot.

**Figure 8:**
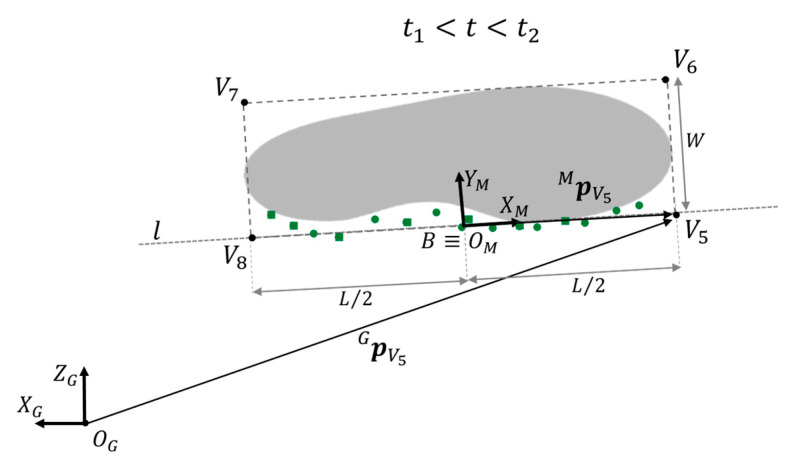
Rectangle approximation of the footprint of the non-instrumented foot during its flat-foot phase. The points detected by the rear (green dots) and front distance sensors (green squares) during the swing of the instrumented foot are linearly interpolated by the line *l*. Point *B* is the centroid of the medial line of the footprint of the non-instrumented shoe. Distance $\overset{-}{V_{5}V_{6}}$ and distance $\overset{-}{V_{5}V_{8}}$ are equal to shoe width *W* and length *L*, respectively. For instance, the position of *V*_5_ with respect to the local coordinate system of the non-instrumented foot (CS_M_), pV5M, is calculated by means of geometrical rules exploiting L/2. Then, the position vector pV5G is computed to describe the point *V*_5_ with respect to the global coordinate system CS_G_.

The line *l* ∈ x_G_-z_G_, representing the medial side of the approximated footprint of the non-instrumented shoe, was obtained through least square fitting using the points contained in the matrix ***M***. In addition, the centroid B of the points was also computed. The local coordinate system of the non-instrumented foot, CS_M_, was then defined with the x-axis aligned with line *l* and centered in B (Figure 8).

The time-invariant positions of vertices $V_{i}$ (*i* = 5, 6, 7, 8) of the rectangle approximating the footprint with respect to CS_M_, pViM (*i* = 5, 6, 7, 8), were defined as follows:

(13) pV5ML20, pV6ML2W, pV7M−L2W, pV8M−L20

where *L* and *W* are the shoe length and width, respectively. 

Then the positions of footprint vertices $V_{i}$ (*i* = 5, 6, 7, 8) with respect to CS_G_, pViG(*i* = 5, 6, 7, 8), were computed:

(14) pViG(t)1=RMGpBG(t)01·pViM1 i=5,6,7,8 t∈[t1,t2]

where pBG is the translation vector between B and O_G_, and RMG is the time-invariant rotation matrix between CS_M_ and CS_G_, defined as

(15) RMG=xG·xMzG·xMxG·yMzG·yM

Since the non-instrumented foot is still during *t*_1_-*t*_2_, its footprint can be defined from the estimated positions of the footprint vertices pViG(t) (*i* = 5, 6, 7, 8) with *t* within *t*_1_ and *t*_2_.

To improve the robustness of the fitting procedure, a data cleaning procedure on the detected points of the medial side of the non-instrumented foot was applied for outlier removal. For further details see Appendix A.

#### 2.2.5. Estimation of the Base of Support Parameters

The position of the footprints of both instrumented and non-instrumented feet were described by the relevant rectangle centroids (C_I_ and C_NI_). The direction of progression of the instrumented foot was identified by the line between the footprint centroids of the instrumented foot, C_I_, between *t*_0_ and *t_f_*.

**Figure 9:**
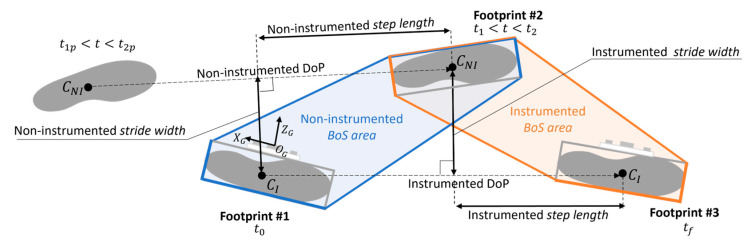
Definitions of base of support (BoS) parameters: BoS area, step length, and stride width. The black dots represent the feet centroids of the instrumented (C_I_) and non-instrumented foot (C_NI_). Dashed lines are the directions of progression (DoP) of instrumented and non-instrumented feet. Blue and orange lines define the outer edges of the instrumented and non-instrumented BoS areas, respectively. *t*_1*p*_ *− t*_2*p*_ and *t*_1_ *− t*_2_ are portions of two consecutive flat-foot phases of the non-instrumented foot. The global coordinate system CS_G_ is taken as the reference system for all the BoS parameters depicted.

Then, the following parameters chosen for the description of the BoS were extracted (Figure 9):

- *BoS area* was defined as the largest area among the ones identified by the footprints vertices of opposite feet including the entire footprint regions. Outer edges of the BoS should not intersect the footprint regions. Rectangle footprints areas were easily calculated, while the irregular area between them was achieved with Bretschneider formula for irregular polygons. The instrumented *BoS area* ended with a contact with the ground of the instrumented foot and it was defined by footprints #2 and #3 (Figure 9).
- *Step length* (coinciding with the BoS length) was identified as the displacement along the direction of progression between a footprint centroid position and the consecutive centroid position of the opposite footprint [3]. Thus, the instrumented *step length* was defined along the direction of progression of the instrumented foot between C_NI_(*t*_1_ − *t*_2_) and C_I_(*t_f_*).
- *Stride width* (coinciding with the BoS width) was determined as the perpendicular distance between a footprint centroid and the direction of progression of the opposite foot [3,4]. Thus, the instrumented *stride width* was defined by C_NI_(*t*_1_ − *t*_2_) and the direction of progression of the instrumented foot.

Considering a generic gait cycle of the non-instrumented foot, all the non-instrumented BoS-related parameters could be similarly estimated. Thus, with reference to two consecutive gait cycles, the *BoS area*, *step length*, and *stride width* of both instrumented and non-instrumented sides were extracted (Figure 9).

For the sake of clearness, the non-instrumented *BoS area* ended with a contact with the ground of the non-instrumented foot and it was defined by footprints #1 and #2. The non-instrumented *step length* was defined along the direction of progression of the non-instrumented foot between C_I_(*t*_0_) and C_NI_(*t*_1_ − *t*_2_). The non-instrumented *stride width* was defined by C_I_(*t*_0_) and the direction of progression of the non-instrumented foot.

### 2.3. Reference Base of Support Parameters Estimation Based on Stereophotogrammetry Data

**Figure 10:**
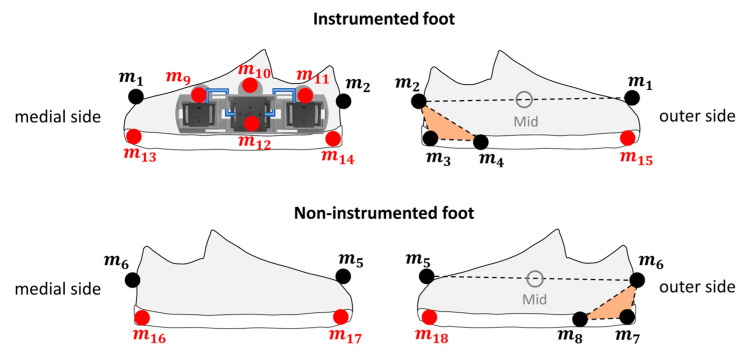
Positions of the 18 retro-reflective markers attached for validation purposes. The right foot was instrumented with the wearable system. The virtual markers in red (*m*_9_–*m*_18_) were used only during the initial standing acquisition for calibration purposes. Mid was the midpoint between heel and toe markers (*m*_1_–*m*_2_ for instrumented foot; *m*_5_–*m*_6_ for non-instrumented foot). The marker clusters highlighted in orange (*m*_2_–*m*_4_ for instrumented foot; *m*_6_–*m*_8_ for non-instrumented foot) defined foot coordinate systems.

For validation purposes, an SP was used to obtain gold standard estimates of the BoS. A total of 18 retro-reflective markers were attached to both feet (*m*_1_–*m*_18_) (Figure 10).

The positions of virtual markers *m*_9_–*m*_15_ on the instrumented foot were calibrated during a preliminary standing trial with respect to the rigid marker cluster defined by *m*_2_–*m*_4_, according to the CAST procedure to avoid visibility issues [33]. Similarly, the positions of virtual markers *m*_16_–*m*_18_ on the non-instrumented foot were calibrated with respect to a rigid marker cluster defined by *m*_6_–*m*_8_ to avoid visibility issues and undesired infrared wave reflections during walking trials. Then, virtual markers *m*_9_–*m*_18_ were removed before recording the walking trials, and their virtual trajectories were reconstructed [33].

Reference initial and final contact instants were estimated using the method proposed by O’Connor et al. [34,35] from the trajectories of the midpoint (Mid) between the heel and toe markers (*m*_1_–*m*_2_ for instrumented foot and *m*_5_–*m*_6_ for non-instrumented foot). The flat-foot instants of both feet were determined based on a parametric zero-velocity detector applied to the norm of the velocity of the foot midpoint [27,28].

The positions of the instrumented and non-instrumented footprint vertices with respect to the SP coordinate system (CS_SP_), pViSP (*i* = 1,…,8), were defined during flat-foot instants from the positions of m_3_ and m_13_-m_15_ (instrumented foot), and *m*_7_ and *m*_16_–*m*_18_ (non-instrumented foot).

To compare BoS parameters as estimated by the wearable system and the SP, the footprints estimated by the SP were expressed in the same global coordinate system of the MIMU (CS_G_). To this purpose, the marker cluster *m*_9_–*m*_12_, rigidly connected with the rigid support, was used to define a marker-based local coordinate system coinciding with CSs and to obtain the MIMU orientation with respect to CS_SP_. By knowing the orientation of the MIMU in both CS_G_ and CS_SP_, it was then possible to obtain the transformation matrix between CS_G_ and CS_SP_. Then, the positions of the footprints vertices could be expressed with respect to CS_G_ (pViG (*i* = 1,…,8)) and the BoS parameters were identified following the definitions adopted for the estimates from wearable system data (see Figure 9).

### 2.4. Experimental Data Collection

**Figure 11:**
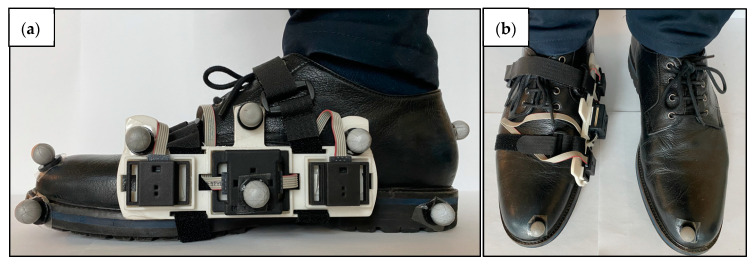
Experimental setup. (**a**) The wearable sensors are attached on a rigid support fixed on the medial side of the right shoe. All retro-reflective markers used, including the virtual ones, are shown. The customized rigid support is optimized to minimize the volume of the wearable system and to host a central MIMU, two lateral distance sensors, and three markers in the upper parts. (**b**) Complete configuration of the experimental setup showing only the markers used for walking trials.

The right foot was instrumented with the wearable system, including an MIMU and two DSs, as shown in Figure 11. The trajectories of retro-reflective markers were recorded by a 12-camera SP system (mod. Vero, Vicon, UK). The wearable system and SP were synchronized using a hardware solution.

Thirteen healthy volunteers (gender: 7F, 6M; age: 25.6 ± 1.8 y.o.) were enrolled. Before starting the experiments, a ten-minute MIMU warm-up was performed to limit the temperature effects on the sensor readings. After a 10-second standing acquisition, the participants were asked to walk along a 5 m straight path on level ground 10 times at 3 different self-selected speeds (slow, comfortable, and fast).

Experiments conformed to the standards set in the Declaration of Helsinki. This protocol was reviewed and approved by the Ethics Committee of the University Hospital of Cagliari (Prot. PG/2021/1195) and participants provided their written informed consent to participate in this study. 

### 2.5. Method Performance Assessment and Statistical Analysis

For each subject, the three tested speeds (slow, comfortable, and fast) were analyzed separately. Right and left estimates were averaged assuming the symmetry of healthy gait.

For each single estimate of each BoS parameter (*step length*, *stride width*, and *BoS area*), errors were computed as the difference between values provided by the wearable system and the reference SP. Subsequently, for each speed level, mean errors (ME), root mean square errors (RMSE), and mean absolute percentage errors (MAE%) were calculated over the 10 trial repetitions and over subjects.

In addition, mean values of the estimated parameters (MV) were calculated over the 10 trial repetitions and over subjects separately for each speed.

The agreement between wearable system and SP estimates was quantified by performing a Bland–Altman analysis with limits of agreement at 95% (LoA) and by calculating the Pearson correlation coefficient (*r_xy_*).

**Figure 12:**
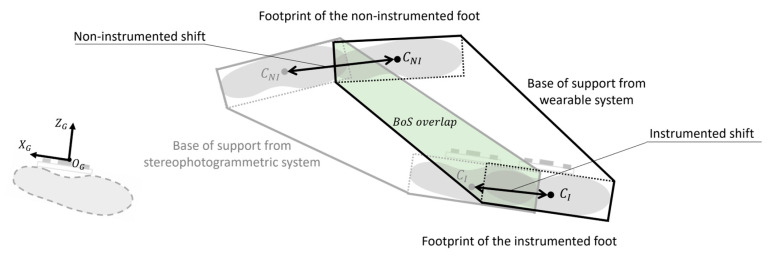
Metrics to describe the base of support estimation. In this example the instrumented base of support areas from the wearable system (black) and stereophotogrammetric system (grey) are illustrated. The base of support overlap (green) is defined as the percentage of the total base of support area shared by sensor-based area and the one obtained from the stereophotogrammetric system. Instrumented and non-instrumented shifts are the distances between footprints’ centroids of instrumented (*C_I_*) and non-instrumented foot (*C_NI_*) calculated with the wearable system and the stereophotogrammetric system.

Errors affecting the BoS estimation were evaluated in terms of errors on the estimation of the area positioning (*BoS overlap%*) (Equation (16)) and errors on footprint positioning (*footprint shift*) (Equation (17)), as shown in Figure 12:

- *BoS overlap%:*

(16) $$BoS\;overlap\%=\frac{BoS\;overlap}{BoS\;Area_{SP}}\times 100$$

where BoS overlap is the area shared by BoS from the wearable system and from SP (*BoS Area_SP_*). Thus, a perfect overlap would be equal to 100%.

- *Footprint shift* was defined as the distance between the footprints’ centroids of the same foot calculated with the wearable system and SP:

(17) $$Footprint\;shift=\overset{-}{C_{WS}C_{SP}}$$

where $C_{WS}$ and $C_{SP}$ are the footprint centroids calculated with the wearable system and SP, respectively. *Footprint shifts* were analyzed on the ground floor (plane x_G_-z_G_), thus *footprint shift_x_* and *shift_z_* were computed.

The analysis was conducted separately for the instrumented and non-instrumented side. We expected that the footprint positioning of the non-instrumented foot would be affected by larger errors than the instrumented one, since its position was only determined by the detection of its medial side by the DS.

Preliminary Shapiro–Wilk tests of normality on the different types of error distributions were carried out to select the most appropriate subsequent statistical analysis.

To assess the effect of speed on BoS parameters estimation (normal distributions), a one-way repeated measure analysis of variance (ANOVA) was performed. Conversely, to assess the effect of speed and differences between instrumented/non-instrumented sides on *BoS overlap%* and *footprint shift* (non-normal distributions), a 3 × 2 Friedman test was used. The significance for all statistical tests was determined at *p* < 0.05 and a Bonferroni adjustment was used to determine statistical significance.

## 3. Results

The number of strides analyzed for each tested walking speed and the average speed values are shown in Table 1.

For each BoS parameter, a description of the method performance is presented in Table 2.

**Figure 13:**
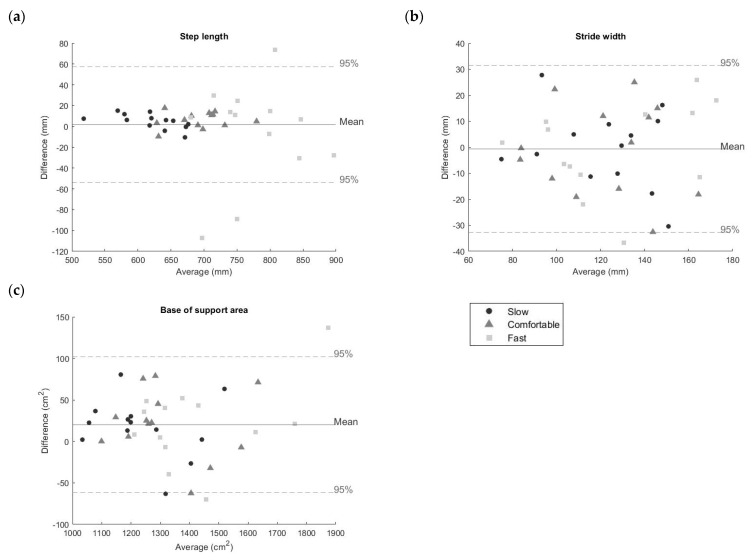
Bland–Altman plots showing the agreement between base of support parameters estimates ((**a**) step length, (**b**) stride width, and (**c**) base of support area) computed with the wearable system and the stereophotogrammetric system. Black dots represent slow speed, grey triangles represent comfortable speed, and light grey squares represent fast speed. Mean values and 95% limits of agreement were calculated across all speeds. The plots include a point for each subject considering separately each walking speed, thus 39 points (3 speeds × 13 subjects) are depicted.

For each parameter, Bland–Altman plots are reported in Figure 13.

According to the Shapiro–Wilk tests, mean errors of BoS parameters (*step length*, *stride width*, and *BoS area*) were normally distributed. No significant differences across walking speeds were found (*p* > 0.05).

The instrumented *BoS overlap%* at slow, comfortable, and fast speed was equal to 75.7 ± 4.3%, 73.9 ± 5.4%, and 70.7 ± 7.3%, respectively. While the non-instrumented *BoS overlap%* at slow, comfortable, and fast speed was equal to 89.1 ± 3.5%, 88.8 ± 4.3%, and 88.7 ± 3.3%, respectively. According to the Shapiro–Wilk test, *BoS overlap%* values were not normally distributed. Statistically significant differences were found between instrumented and non-instrumented *BoS overlap%* values for each speed (*p* < 0.05). A significant difference was found across speeds in the *BoS overlap%* only for the instrumented side between slow and fast speeds (*p* < 0.05).

**Figure 14:**
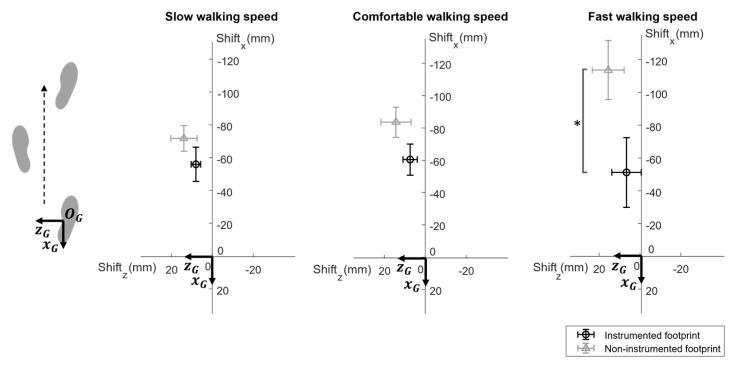
Mean plots with standard deviation bars for footprint shift_x_ with respect to footprint shift_z_ for the different walking speeds. The footprint shifts of the instrumented foot (black) and non-instrumented foot (grey) are the distances on the ground plane (x_G_-z_G_) between sensor-based footprint centroids and the ones obtained with the stereophotogrammetric system. * statistically significant difference at *p* < 0.05.

Results showed that *footprint shift* values for the non-instrumented foot were larger than those found for the instrumented foot for each walking speed (Figure 14). According to the Shapiro–Wilk test, *footprint shift* values were not normally distributed. Statistically significant differences were found between instrumented and non-instrumented *footprint shift_x_* values for fast speed (*p* < 0.05). A significant difference was found across speeds in the *footprint shift_x_* only for the non-instrumented foot between slow and fast speeds (*p* < 0.05). No significant differences were found pairwise comparing results of *footprint shift_z_* values (*p* > 0.05).

## 4. Discussion

In this study, an original method for the stride-by-stride estimation of the BoS and related parameters, such as right and left *step length*, *stride width*, and *BoS area*, is presented, and validated on gait data recorded on 13 young healthy adults at different speeds. 

The unique feature of the proposed method is that it requires to instrument one foot only, thus improving system wearability with respect to previous solutions requiring equipping both feet with an emitter and a receiver [15,16,18,19].

The validity of the estimated BoS parameters was assessed on more than 1900 strides against a gold standard (SP) and *strong* to *very strong* correlations were found for all parameters at every walking speed (0.8 $<r_{xy}<$ 0.98).

The RMSE values affecting *stride width* estimates varied from slow (~0.90 m/s) to high speed (~1.51 m/s) between 14 and 18 mm (MAE% = 9.4% to 11.5%), whereas the RMSE values affecting the *BoS area* varied between 39 and 52 cm^2^ (MAE% = 2.5% to 2.8%). No significant differences on the estimation errors were found for different walking speeds (*p* > 0.05).

The RMSE values for the *step length* estimation were equal to 10 mm (MAE% = 1.2% to 1.4%) at slow and comfortable (~1.17 m/s) speeds. By increasing the walking speed to ~1.51 m/s, the accuracy in the detection of the *step length* worsened, resulting in an RMSE equal to 46 mm (MAE% = 4.1%). However, despite the larger errors found during fast walking, these differences among speeds were not statistically significant (*p* > 0.05).

LoA intervals and the visual inspection of the Bland–Altman plots confirmed a slight worsening in the agreement between SP and the wearable system at high speed, which could be associated with a lower number of data points recorded by DS sampling at 50 Hz and the expected lower accuracy in the orientation estimation [36,37].

It can be assumed that the use of DS with higher sampling frequency, allowing an increase in the number of detected points, would improve the identification of the non-instrumented foot and, consequently, the estimation of BoS parameters.

As expected, errors in the estimation of the position of the footprint of the non-instrumented foot were larger than those affecting the instrumented foot (absolute averaged *footprint shift_x_* = 90 mm vs. 56 mm) with a significant difference at fast speed (*p* < 0.05). This difference is due to the different procedures implemented to estimate the feet position: the non-instrumented foot was identified by scanning its medial side using the DSs, whereas the position of the instrumented foot was determined by double integrating its accelerations.

Similarly, the *BoS overlap%* values between sensor-based and SP-based *BoS areas* were significantly larger for the non-instrumented side than the instrumented one (averaged *BoS overlap%* = 88.9% vs. 73.4%, *p* < 0.05). In fact, as shown in Figure 9, the estimation of the non-instrumented BoS was only affected by errors in the position of the estimated footprint of the non-instrumented foot (#2). In fact, the position of the previous footprint of the instrumented foot (#1) was made to coincide with the origin of the CS_G_, and therefore set equal to zero at every gait cycle. Conversely, the estimation of the instrumented BoS was corrupted by both errors in the position of the non-instrumented foot (#2) and the position of the footprint of the instrumented foot (#3). A possible solution to overcome this problem could be the implementation of a time reversal and inverting the reference initial foot position.

In general, errors affecting the estimation of *step length* and *stride width* at comfortable speed were comparable or lower than those reported by Weenk and colleagues (MAE on *step length* = 17 mm; MAE on *stride width* = 12 mm) [16].

To the best of authors’ knowledge, there are no previous studies computing the BoS in terms of area during walking using wearable technologies against which to compare our results.

The proposed method comes with some limitations. First, it should be noted that, in contrast with previous studies instrumenting both feet [16,18,19], our method does not provide the trajectory and orientation of the non-instrumented foot over time, but only its footprint position and orientation. 

Second, the method performance was validated on normal and almost straight walking, and caution should be paid to extend results to the analysis of curvilinear gait and to patients exhibiting abnormal gait patterns mixed of unpredictable accelerations and decelerations in walking speed with superimposed foot twisting, such as choreiform gait. In real world conditions, the non-instrumented foot might not be detected, or detected with a lower accuracy, during sharp turns, obstacles avoidance, or walking on uneven terrains. In these cases, a solution to increase method robustness would be to attach an additional IMU on the non-instrumented foot in order to track its trajectory during the strides for which inter-foot distance data are missing.

Lastly, it should be acknowledged that, due to the positioning of the wearable system on the medial side of a shoe, the minimum inter-foot distance to avoid the collision between feet must be greater than the system thickness (i.e., 22 mm). We did not encounter any problems when analyzing the gait of healthy subjects and most neurological disorders lead to a wider-based gait [38]. However, it cannot be excluded that this could be an issue in parkinsonian patients typically showing a normal to narrow base of support [39].

## 5. Conclusions

This study described and validated a wearable system and a novel method for the estimation of BoS parameters such as step length, stride width, and BoS area, providing valuable and accurate information for a complete gait analysis and dynamic stability investigation.

The designed system is lightweight, it only requires instrumenting a single shoe, and it can be suitable for acquisitions in free-living contexts.

Overall, results on healthy subjects were very promising, suggesting that the proposed system and the associated algorithms may provide an accurate and valid solution for the dynamic estimation of BoS parameters, expanding the possibilities to investigate dynamic balance also outside the laboratory setting.

Future work should focus on experimental sessions analyzing more complex locomotion tasks including turning, clinical tests, and simulated daily activities. In addition, the accuracy and the robustness of the proposed system should be investigated on subjects suffering from movement disorders.

## Figures and Tables

**Table 1:** Number of analyzed strides and averaged walking speed.

	Slow	Comfortable	Fast
**Stride number**	790	778	355
**Walking speed (m/s)**	0.90 ± 0.14	1.17 ± 0.14	1.51 ± 0.20

**Table 2:** Results of base of support parameters: mean values (MV) and differences with respect to stereophotogrammetric system. ME = mean error, RMSE = root mean squared error, MAE = mean absolute error, *r_xy_* = Pearson correlation coefficient, LoA = limits of agreement at 95%.

		Slow	Comfortable	Fast
**Step length**	MV ± SD (mm)	618 ± 43	687 ± 41	753 ± 65
	ME ± SD (mm)	1 ± 11	−1 ± 11	−24 ± 41
	RMSE (mm)	10	10	46
	MAE (%)	1.37	1.23	4.12
	$r_{xy}$	0.979	0.968	0.802
	LoA (mm)	−20 to 22	−22 to 20	−100 to 57
**Stride width**	MV ± SD (mm)	123 ± 23	124 ± 25	124 ± 36
	ME ± SD (mm)	0 ± 15	0 ± 17	0 ± 19
	RMSE (mm)	14	16	18
	MAE (%)	9.44	10.8	11.45
	$r_{xy}$	0.835	0.801	0.857
	LoA (mm)	−29 to 30	−32 to 34	−37 to 36
**Base of support area**	MV ± SD (cm^2^)	1246 ± 148	1238 ± 160	1434 ± 218
	ME ± SD (cm^2^)	17 ± 36	21 ± 42	22 ± 50
	RMSE (cm^2^)	39	45	52
	MAE (%)	2.53	2.78	2.74
	$r_{xy}$	0.972	0.967	0.976
	LoA (cm^2^)	−54 to 88	−61 to 103	−75 to 119

**Figure A1:**
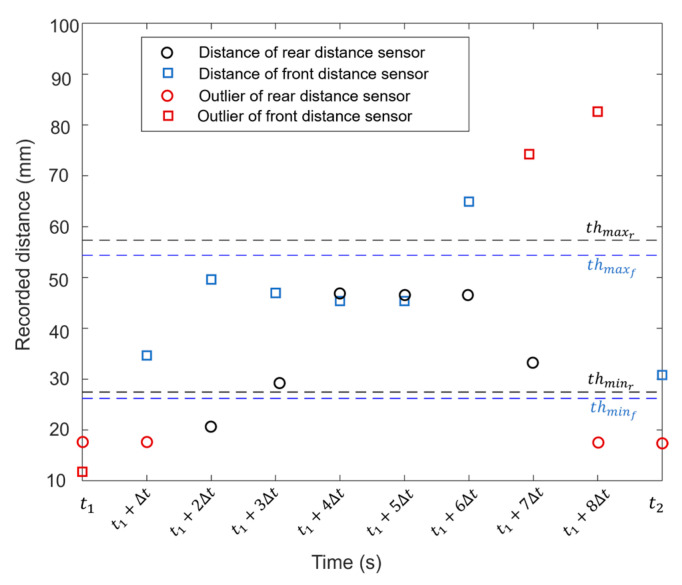
Example of recorded distance data from rear (dots) and front (squares) distance sensors during a swing of the instrumented foot during a walking trial at fast speed (1.8 m/s). Red data correspond to the outliers exceeding the 95% confidence intervals. The dashed horizontal lines determine the thresholds within which the distance data from rear (black) and front (blue) distance sensors are considered reliable.

## Data Availability

The Matlab code used in this project can be provided upon request to the corresponding author.

## Abbreviations

This short glossary provides the meaning of the main abbreviations used in this work:

BoS	base of support
MIMU	magneto-inertial measurement unit
Infrared time-of-flight	infrared time of flight
SP	stereophotogrammetric system
DS	distance sensor(s)
***f***(t)	specific force
***a***(t)	acceleration
*d_f_*(t)	distance recorded by the front DS attached to the instrumented shoe
*d_r_*(t)	distance recorded by the rear DS attached to the instrumented shoe
CS_S_	MIMU coordinate system
CS_E_	Earth coordinate system
CS_G_	global coordinate system
CS_SP_	SP coordinate system
*t*_0_ and *t_f_*	flat-foot instants of the instrumented foot that determine the beginning and the end of the considered generic gait cycle
*t*_1_ and *t*_2_	first and last instants in which at least a DS records a distance different from zero within the same gait cycle
*V*_1_, *V*_2_, *V*_3_, and *V*_4_	vertices of the footprint of the instrumented foot
*V*_5_, *V*_6_, *V*_7_, and *V*_8_	vertices of the footprint of the non-instrumented foot
*R*	generic point of the medial side of the non-instrumented foot detected by the front DS
*F*	generic point of the medial side of the non-instrumented foot detected by the rear DS

## Appendix A

In this appendix, the procedure followed to remove the distance outliers recorded by the DS is presented.

During the scanning of the non-instrumented foot, the presence of distance outliers was observed. Since the non-instrumented foot was still during the detection of its medial side by the DS, it was reasonable to assume that the recorded distances should not exhibit great changes. However, sharp variations of distance data often occurred, especially at the beginning and at the end of the scanning, because they were not consistent with the true shape of the detected medial side of the non-instrumented foot. These outliers were assumed to correspond to the detection of points belonging to the ground, or the shank, or to stochastic reflection due to surface discontinuities.

To remove the unreliable distance data, the following method was implemented (Figure A1):

(1) Preliminary removal of 95% outliers: for each swing of the instrumented foot and for each DS, observations exceeding the 95% confidence interval of the recorded distances were excluded;
(2) Removal of less reliable data points: to define distance thresholds, the observations within the 95% confidence interval were averaged to compute the mean distance values from rear DS ($\overset{-}{d_{r}}$) and front DS ($\overset{-}{d_{f}}$). Then, empirical thresholds equal to $\overset{-}{d_{r}}$ ± 35%·$\overset{-}{d_{r}}$ and $\overset{-}{d_{f}}$ ± 35%·$\overset{-}{d_{f}}$ were calculated to define the interval in which the distance data were considered reliable. If a distance value exceeded the range determined by these thresholds, then it was excluded from further analyses. Observations below the lower threshold th_min_ ($\overset{-}{d_{r}}$ − 35%·$\overset{-}{d_{r}}$ for distances from rear DS, and $\overset{-}{d_{f}}$ − 35%·$\overset{-}{d_{f}}$ for distances from front DS) probably detected the ground, while observations larger than the higher threshold th_max_ ($\overset{-}{d_{r}}$ + 35%·$\overset{-}{d_{r}}$ for distances from rear DS, and $\overset{-}{d_{f}}$ + 35%·$\overset{-}{d_{f}}$ for distances from front DS) could be associated with distances from the shank.
